# Robotic spine surgery - Surgical technique and nuances for improving safety

**DOI:** 10.37796/2211-8039.1644

**Published:** 2025-03-01

**Authors:** Mun Chun Lai, Pirateb Paramasivam Meenakshi Sundaram, Arun-Kumar Kaliya-Perumal, Jacob Yoong-Leong Oh

**Affiliations:** a Division of Spine, Department of Orthopaedic Surgery, Tan Tock Seng Hospital, Singapore; b Rehabilitation Research Institute of Singapore, Lee Kong Chian School of Medicine, Nanyang Technological University, Singapore

**Keywords:** Computer-assisted surgeries, Image guided surgery, Patient safety, Robotics, Spinal spondylosis

## Abstract

The integration of robotic technology into spinal surgery has led to a remarkable transformation, offering improved precision and safety. The “CT-Fluoro merge” and “Scan-and-Plan” methods for pedicle screw application promise numerous advantages, minimizing risks associated with traditional approaches. Our early series of patients who underwent robot-assisted pedicle screw placements for various indications, predominantly lumbar spondylosis, revealed no complications related to screws, neurological deficits, or unplanned returns to the operating theatre, thus emphasizing the safety and effectiveness of the robot. Studies have consistently demonstrated the superiority of robot-assisted pedicle screw placement in terms of accuracy, with decreasing rates of technical issues indicating improvements in reliability and precision. Our experiences align with these findings, signalling the emergence of robotic spine surgery as the forthcoming standard of care in the field. This short communication explores the steps involved in robotic pedicle screw placement, discusses nuances for improving safety, and emphasizes its benefits and implications for the future of spinal care.

## 1. Introduction

In recent years, the field of spinal surgery has undergone a remarkable transformation with the introduction of robotic technology, marking the fastest growth within the orthopaedic domain. While traditional methods, especially non-real-time navigation-based pedicle screw application, have limitations regarding accuracy, computer navigation and the integration of robotic assistance offer numerous advantages, such as enhanced precision, less invasiveness, faster recovery, and improved pain management [1–3]. These benefits would not have been possible to the same extent without the use of robots. As demand rises, robotic systems are becoming more common. While initially expensive, they could lead to long-term savings, especially when their usage is high. At our institution, we use the Mazor X Stealth Edition Robot (Medtronic Inc., Dublin, Ireland), known for its operational flexibility, offering two distinct methods: CT-Fluoro merge and Scan-and-Plan [4,5]. The CT-Fluoro merge combines preoperative CT imaging with intra-operative fluoroscopy, enhancing pre-surgical planning. In contrast, the Scan-and-Plan method allows for real-time adjustments during surgery based on fluoroscopic imaging. Each method offers unique advantages, allowing for adaptation to different surgical needs, including the complexity of the procedure, patient anatomy, and intraoperative findings.

## 2. Methods

This study is based on an early series of 56 patients (27 males, 29 females; median age 69.5 years) who underwent robot-assisted pedicle screw placement using the Mazor X Stealth Edition Robot for lumbar stabilization and fusion. Most patients presented with degenerative lumbar spinal stenosis, except for one trauma case. Surgical approaches included 7 open surgeries and 49 minimally invasive procedures.

The CT-Fluoro merge method was used in 51 cases, while the Scan-and-Plan method was employed in 5 cases. The procedures varied in complexity, consisting of 31 single-level, 19 two-level, and 6 threelevel surgeries. A total of 308 pedicle screws were applied with robotic assistance. The CT-Fluoro merge method was predominantly used, preferred for its ability to facilitate pre-surgery planning a day in advance, thereby reducing surgical duration. In addition, this method allows the robot to recognize each vertebra as a distinct segment, regardless of the patient’s position, while the intraoperative O-arm-based Scan-and-Plan method treats the spine as a single segment.

**Fig. 1:**
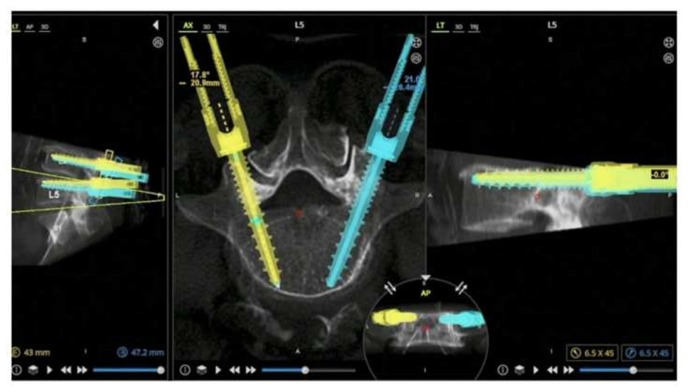
Preop CT planning of the pedicle screws in axial and sagittal view.

**Fig. 2:**
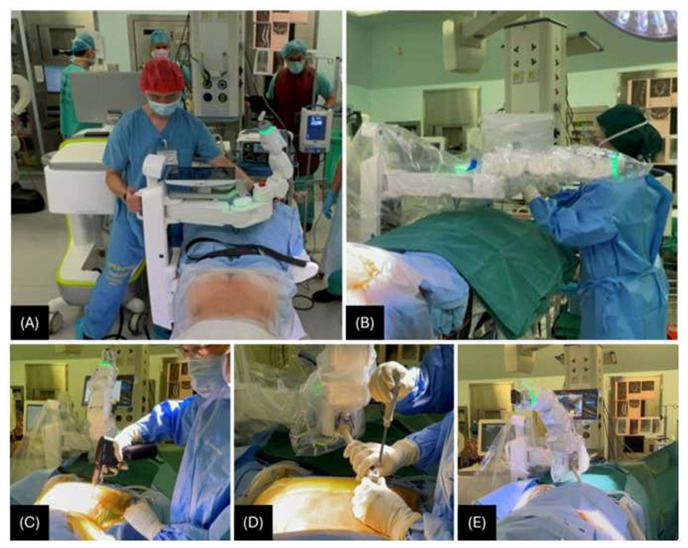
A) Table mounting; B) Sterile draping of the robotic arm; C) Schanz screw being inserted in the posterior superior iliac spine (PSIS); D) Patient mounting - attaching the robot to the patient (Schanz screw); E) Topographical scan of the surgical field to allow the robotic arm to execute efficient manoeuvring.

**Fig. 3:**
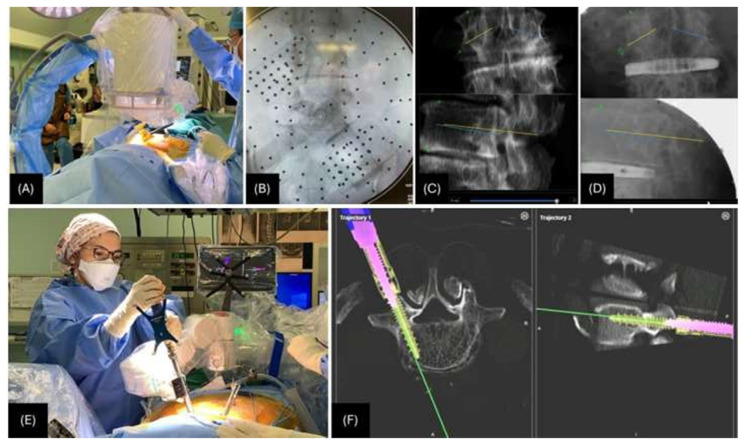
A) Fluoroscopic imaging, both AP and Oblique views to perform the CT-Fluoro merge; B) Illustration of the AP view in fluoroscopy with reference markers within the area of interest; C, D) CTFluoro merge; C) Preoperative CT, note the bony landmarks (green dots) and pedicle tracts (lines) marked by the robotic system; D) Post-oblique lateral interbody fusion (OLIF) fluoroscopy along with markers to execute the merge; E) Pedicle screw insertion utilizing the robotic arm guidance; F) Navigation monitor view.

The CT-Fluoro merge unfolds in six distinct stages: two pre-operative and four intraoperative [5]. The pre-operative focus is on performing a CT scan and planning the optimal diameter, size, and trajectory of the pedicle screws in all three planes (coronal, sagittal, and axial) (Fig. 1), while the subsequent intraoperative stages involve the implementation of the surgical procedure. Firstly, the robotic arm unit is mounted to the operating table via a bed frame adapter and positioned to the patient using a bone mount bridge (Fig. 2A). Before the surgery begins, the robotic arm unit is sterilely draped, with the reference frame and arm guide securely attached (Fig. 2B). The robot is then connected to the patient using a single percutaneously inserted Schanz screw in the posterior superior iliac spine (PSIS) as a reference point (Fig. 2C and D). This is followed by a topographical scan of the surgical field to allow the robotic arm to execute efficient manoeuvring (Fig. 2E). Anterior-to-posterior and oblique-to-lateral fluoroscopic radiographs are taken (Fig. 3A and B). These images are subsequently registered to the robot and merged with the pre-operative CT image to precisely execute the surgery as planned (Fig. 3C and D). The robotic arm moves precisely along the pre-determined trajectory to facilitate the application of screws. Navigational tools are then inserted through the robotic arm into the pedicle to prepare it for screw application, while the arm maintains a steady trajectory. Finally, the screws are inserted with the robotic arm’s guidance (Fig. 3E and F).

## 3. Results

Upon evaluating the surgical outcomes in patients who underwent pedicle screw placements using the robot, no screw-related complications, unplanned returns to the operating room, or postoperative neurological deficits were observed, demonstrating the safety of robotic spine surgery. It is worth noting a few additional instances that emerged outside the scope of the 56 patients discussed above. These instances demonstrate the practical challenges of robot-assisted surgeries, resulting in the abandonment of the robot in four cases and emphasizing the need for further refinement in technology and procedural integration. The reasons being software registration failures due to poor image quality from either the C-arm or O-arm in two patients, and a robotic arm malfunction in one instance, where the arm became entrapped under the sterile drape. In another minimally invasive transforaminal lumbar interbody fusion (MIS-TLIF) case, concerns arose regarding soft tissue retraction, leading to apprehensions about the accuracy of the table-mounted robot. However, the rate of such occurrences remains low. This study (Reference No. 2024-3041) was approved by the Domain Specific Review Board, National Healthcare Group, Singapore and was conducted in accordance with the Declaration of Helsinki.

## 4. Discussion

**Fig. 4:**
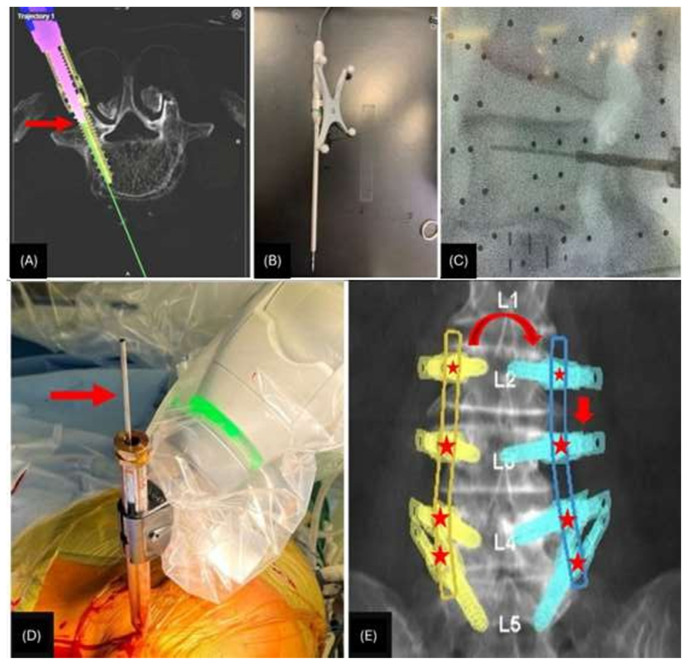
A) Depicts tactile feedback, emphasizing that when any of the navigated instruments is on bone (arrow), the image displayed on the navigation monitor should correspond accordingly; B) Shows the Midas device (burr), which is utilized to prepare the pedicle; C) Highlights the use of supplementary fluoroscopic imaging; D) Demonstrates the straw device (arrow), employed for palpation to confirm the absence of significant breaches; E) Depicts the routine of pre-drilling pilot holes before screw insertion.

Over nearly two decades, studies have consistently shown that robot-assisted techniques are safe and effective, often surpassing the accuracy of traditional freehand methods [6–8]. This is a testament to the technology, as it minimizes the potential for human error by allowing the robot to determine the screw placement and trajectory based on the pre-operative CT scan. Although a learning curve is associated with training on the robotic system, it can be effectively overcome in a relatively short period [9,10]. Drawing from our experiences, we propose 5 key technical tips to enhance safety in robotic pedicle screw placement (Fig. 4). These methods encompass: 1) Tactile feedback integration, wherein the image displayed on the navigation monitor corresponds to palpation of the bony surface using the Midas device (Fig. 4A and B). 2) Utilization of a delicate “Bounce” technique with the Midas device, advancing in 2 mm increments with intermittent retractions, facilitating the perception of pedicle anatomy. Here, it is advised to initiate the Midas device approximately 1–2 cm above the bone to prevent sliding or skiving. 3) Incorporation of supplementary fluoroscopic imaging to ensure an accurate lateral perspective (Fig. 4C). 4) Utilizing the “straw” for palpation to verify the absence of significant breaches (Fig. 4D). 5) Pre-drilling pilot holes before screw insertion, particularly crucial during high-torque manoeuvres in dense bone, to mitigate potential movement in long constructs. Additionally, precautions should be taken to avoid leaning and pushing, which could inadvertently cause the patient to move, as well as refraining from forcefully retracting soft tissue through small incisions. Our experience underscores the safety, feasibility, and accuracy of robotic spine surgery when performed for appropriate indications. With ongoing advancements, robotic spine surgery is poised to become the standard of care in the field.
